# Effect of Tai Chi versus Walking on Oxidative Stress in Mexican Older Adults

**DOI:** 10.1155/2013/298590

**Published:** 2013-07-07

**Authors:** Juana Rosado-Pérez, Rocío Ortiz, Edelmiro Santiago-Osorio, Víctor Manuel Mendoza-Núñez

**Affiliations:** ^1^Unidad de Investigación en Gerontología, Facultad de Estudios Superiores Zaragoza, Universidad Nacional Autónoma de México (UNAM), México, DF, Mexico; ^2^Departamento de Ciencias de la Salud, Universidad Autónoma Metropolitana-Iztapalapa, Apartado Postal 55-535, 09340 México, D.F., Mexico; ^3^Laboratorio de Biología Celular y Molecular del Cáncer, UIDCC, FES-Zaragoza, UNAM, Mexico

## Abstract

It has recently been reported that the practice of Tai Chi reduces oxidative stress (OxS), but it is not clear whether walking or Tai Chi produces a greater antioxidant effect. The aim of the present study was to evaluate the effect of the practice of Tai Chi and walking on markers for OxS. We carried out a quasi-experimental study with 106 older adults between 60 and 74 years of age who were clinically healthy and divided into the following groups: (i) control group (*n* = 23), (ii) walking group (*n* = 43), and (iii) Tai Chi group (*n* = 31). We measured the levels of lipoperoxides (LPO), antioxidant enzymes superoxide dismutase (SOD) and glutathione peroxidase (GPx), and total antioxidant status (TAS) pre- and post-intervention in all subjects. The data were subjected to a covariant analysis. We found lower levels of LPO in the Tai Chi group compared with the walking group (Tai Chi, 0.261 ± 0.02; walking, 0.331 ± 0.02; control, 0.304 ± 0.023 *µ*mol/L; *P* = 0.05). Likewise, we observed significantly higher SOD activity and lower OxS-score in the Tai Chi group (*P* < 0.05). Our findings suggest that the practice of Tai Chi produces a more effective antioxidant effect than walking.

## 1. Introduction

 Aging is a gradual and adaptive process characterized by a relative reduction of the homeostatic response due to the modifications produced by changes inherent to aging and the accumulated impact of the challenges confronted by the organism over the course of its life [1, 2]. In addition to genetic and environmental factors, lifestyles are determining factors for the process and type of individual aging [1]. Although aging is multifactorial and individualized, it has been linked to oxidative stress (OxS), a biochemical alteration that increases the risk of chronic degenerative disease [2, 3].

Physical exercise minimizes the biological changes associated with normal aging, such as reduction in muscle mass, strength, and rapidity of muscle contraction. It also has a positive effect on mitochondrial function, oxidizing enzymatic capacity, aerobic capacity, cardiac contractibility, rapidity of nerve conduction, and glucose intolerance. There is consistent evidence that exercise affects psychological wellbeing, increases longevity, and diminishes the risk of chronic degenerative diseases in old age [4–6]. 

On the other hand, moderate exercise is indicated for maintaining health and preventing chronic diseases in old age. In this respect, walking is one of the most recommended exercises, and it has been shown to have positive effects on various biochemical markers, including those relating to oxidative stress [7–11]. Tai Chi is another modality of exercise that is broadly recommended for the elderly, particularly for preventing falls and psychological wellbeing [12, 13]. In addition, it has recently been observed that Tai Chi stimulates the endogenous antioxidant enzymes activity and decrease the oxidative stress in middle-aged adults and pre- and postmenopausal women [14, 15]. On the other hand, our research group found that Tai Chi diminishes the oxidative stress in older adults [16]. However, it is unknown whether Tai Chi or walking produces a more efficient antioxidant effect. The aim of the present study was to determine the effect of walking, in comparison with Tai Chi, on oxidative stress (OxS) in older Mexican adults. 

## 2. Methods

### 2.1. Design and Subjects

**Figure 1:**
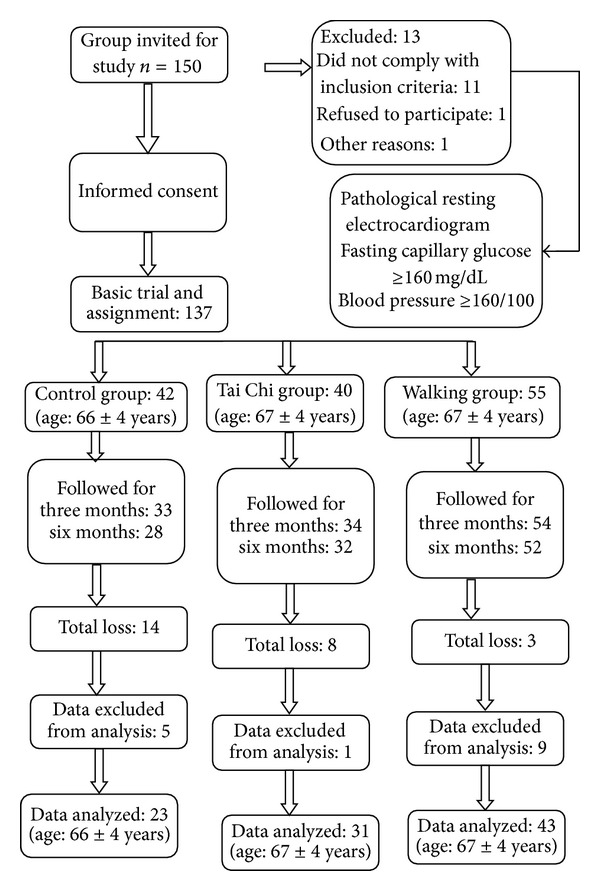
General scheme for study tracking.

We carried out a quasiexperimental study on a sample of 106 seniors who were divided into the following groups: (i) control group (*n* = 23), (ii) Tai Chi group (*n* = 31), and (iii) walking group (*n* = 43). In Figure 1, we outline the study. The criteria for inclusion were as follows: age greater than 60 but less than 74 years, no participation in any physical training program in the 6 months prior to the intervention, free of chronic diseases, normal resting electrocardiogram, and no consumption of antioxidants. The Tai Chi and walking were performed for 6 months in 1-hour daily sessions. The hour included 10 minutes of warmup, 40 minutes of exercise, and 10 minutes of cooldown. All participants exercised with their assigned modalities under the supervision of an expert instructor and personal doctor. Prior to the start of the 6-month study period, the Tai Chi group participated in a three-week period of physical training to work on the basic movements of Tai Chi in order to master forms 8 and 16 of the Yang style [17]. 

### 2.2. Anthropometric and Blood Pressure Measurements

After clinical history and physical examination were conducted, we performed the following anthropometric measurements: weight was measured while the subject was wearing underwear and a clinical smock and in a fasted state (after evacuation). A Torino scale (Tecno Lógica, Mexicana, México, TLM) was used, calibrated before each weight measurement. Height was obtained with an aluminum cursor stadiometer graduated in millimeters. The subject was barefoot, back, and head in contact with the stadiometer in Frankfurt horizontal plane. Body mass index (BMI) was calculated by dividing weight (in kilograms) by height (in squared meters). Waist circumference was measured with a tape measure to the nearest 0.5 cm at umbilical scar level in centimeters. 

Blood pressure was measured in both arms 3 times in the morning, in a fasting condition or 2 hours after breakfast, in sitting and standing positions. A mercurial manometer was used to measure the blood pressure. Subjects with pseudohypertension were identified by applying the Osler technique, which is, feeling the radial pulse when the manometer registered values above the true systolic pressure. Blood pressure was taken by medical technicians who had attended training sessions to standardize the procedures. The technicians were supervised to avoid possible biases in measurement.

### 2.3. Biochemical Analysis

For the biochemical tests, we sampled blood from all participants via venipuncture before the beginning of the exercise program (basal) and after six months of intervention. The samples were obtained between 8 and 9 AM with a previous fasting period of 8 hours and collected in vacuum tubes (Becton-Dickinson, México), without anticoagulant for the biochemical tests (glucose and lipid profile) and with heparin for the tests for OxS. Biochemical analyses (glucose and lipid profile) were conducted using a colorimetric technique in an Autoanalyzer Vitalab Eclipse Merck (Dieren, The Netherlands) DL concentration was calculated using the Friedewald formula.

### 2.4. Plasma TBARS

The TBARS assay was prepared as described by Jentzsch et al. (1996) [18]. In the TBARS assay, one molecule of malondialdehyde reacts with two molecules of thiobarbituric acid (TBA) and thereby produces a pink pigment with absorption peak at 535 nm. Amplification of peroxidation during the assay is prevented by the addition of the chain-breaking antioxidant, butylated hydroxytolueno toluene (BHT). 

### 2.5. Plasma Total Antioxidant Status (TAS)

Antioxidant quantification was done using 2,2′-azino-bis (3-ethylbenzthiazoline-6-sulfonic acid) (ABTS^+^) radical formation kinetics (Randox Laboratories, Ltd., Crumlin Co., UK). The antioxidants present in plasma suppressed the bluish-green staining of the ABTS^+^ cation, which was proportional to the antioxidant concentration level. The kinetics were measured at 600 nm with UV-spectrophotometer Shimadzu UV-1601 (Kyoto, Japan).

### 2.6. Red Blood Cell Superoxide Dismutase (SOD)

The method uses xanthine and xanthine oxidase (XOD) to generate superoxide radicals, which react with 2-(4-iodophenyl)-3-(4-nitrophenol)-5-phenyltetrazolim chloride to form a red formazan dye. SOD activity was measured by degree of inhibition of the reaction (Randox Laboratories Ltd., Crumlin Co., UK). The kinetics were measured at 505 nm with UV-spectrophotometer Shimadzu UV-1601 (Kyoto, Japan). 

### 2.7. Red Blood Cell Glutathione Peroxidase (GPx)

In the presence of glutathione reductase and NADPH, the oxidation of glutathione (GSH) by cumene hydroperoxide is catalyzed by GPx. Oxidized glutathione (GSSG) is immediately converted into the reduced form with a subsequent oxidation of NADPH to NADP+ (Randox Laboratories, Ltd., Crumlin Co., UK). Decrease in absorbance was measured at 340 nm with UV-spectrophotometer Shimadzu UV-1601 (Kyoto, Japan). 

### 2.8. Oxidative Stress Score

Alternative cut-off values of each parameter were defined on the basis of the 90th percentile of young healthy subjects: lipid peroxidation (LPO) ≥ 0.340 mmol/L, superoxide dismutase (SOD) ≤ 170 IU/L, glutathione peroxidase (GPx) ≤ 5500 IU/L, total antioxidant status (TAS) ≤ 0.9 mmol/L, SOD to GPx ratio (SOD/GPx) ≥ 0.023, and antioxidant GAP (GAP) ≤ 190 mmol/L. A stress score (SS) ranging from 1 to 6, representing the severity of biomarkers modifications; a score 1 was given to each value higher or lower than the cutoff. We categorize subjects as follows: according to their scale in SS: without OxS if SS was 0, Slight OxS if SS was 1-2, moderate OxS if SS was 3-4, and severe OxS if SS was 5-6. We have two categories: the subjects without OxS when a stress score (SS) are ranging from 0–2 and subjects with OxS when a stress score (SS) are ranging from 3–6 [19].

### 2.9. Statistical Analysis

The data were analyzed using the SPSS 16.0 (SPSS Inc., Chicago, IL, USA) statistical package. We used descriptive measures, average and standard deviation, and pre- and postintervention data. The data were analyzed using ANOVA, and parameters that displayed significant pre- and postintervention were subjected to multiple covariant analysis using the basal parameter conditions as covariables. 

## 3. Results

### 3.1. Blood Pressure and Biochemical Characteristics

A statistically significant decrease in systolic blood pressure in the subjects who practiced Tai Chi was observed with respect to the control and walking groups (*P* < 0.001) (Table 1).

With regard to biochemical parameters, we found a significant reduction in total cholesterol (baseline, 201 ± 48 versus postintervention, 180 ± 41 mg/dL; *P* < 0.05) and LDL cholesterol (baseline, 116 ± 45 versus postintervention, 99 ± 37 mg/dL; *P* < 0.001) in the subjects who practiced Tai Chi with respect to the control and walking groups (Table 2).

### 3.2. Oxidative Stress Markers

In LPO levels, we found a significant decrease in the Tai Chi group (baseline, 0.287 ± 0.01 versus postintervention, 0.257 ± 0.09 *μ*mol/L; *P* < 0.05); in contrast, there was a significant increase in the walking group (baseline, 0.244 ± 0.01 versus postintervention, 0.330 ± 0.01 *μ*mol/L; *P* < 0.001). Likewise, an increase of SOD activity in the Tai Chi group was observed (baseline, 169 ± 80 versus postintervention, 178 ± 90 U/mL; *P*< 0,05); nevertheless, walking group did not show a significant change postintervention. With regard to GPx activity, the walking group showed a significant increase (baseline, 7756 ± 2697 versus postintervention, 11600 ± 6779 U/L, *P* < 0.001); in contrast there was not a significant increase in the Tai Chi group (baseline, 6225 ± 2869 versus postintervention, 8410 ± 4084 U/L, *P* > 0.05). On the other hand, we found a significant decrease in the OxS-score in the Tai Chi and walking groups, although the change was more evident in the Tai Chi group (Table 3). 

### 3.3. Changes in Oxidative Markers by Intervention

**Figure 2:**
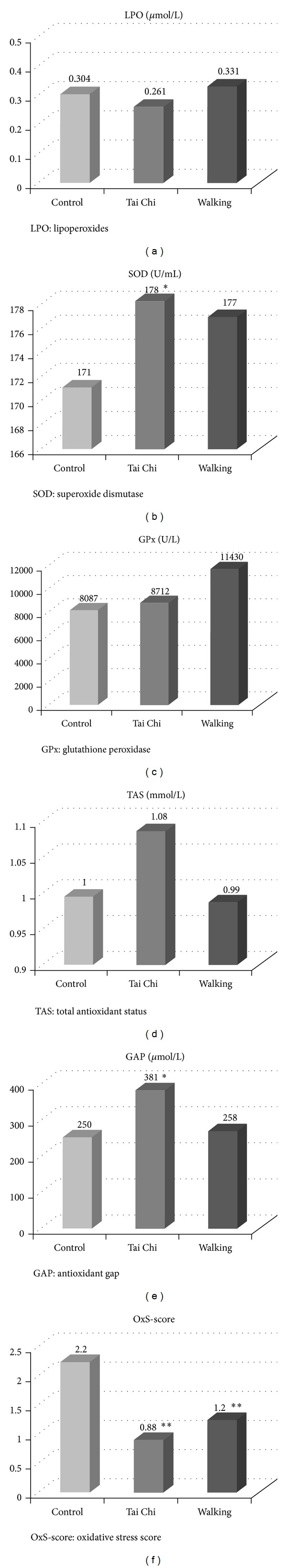
Bars show mean adjusted for initial conditions (ANCOVA Test). (a) LPO: control 0.304 ± 0.023, Tai Chi 0.261 ± 0.02, and walking 0.331 ± 0.02 *μ*mol/L, *P* = 0.08; (b) SOD: control 171 ± 2, Tai Chi 178 ± 2, and walking 177 ± 1 U/mL,*P < 0.05; (c) GPx: control 8087 ± 1113, Tai Chi 8712 ± 923, and walking 11430 ± 711 U/L, *P* = 0.07; (d) TAS: control 1.0 ± 0.035, Tai Chi 1.08 ± 0.029, and walking 0.99 ± 0.22 mmol/L, *P* > 0.05; (e) GAP: control 250 ± 46, Tai Chi 381 ± 37, and walking 258 ± 31 *μ*mol/L, *P* < 0.05; (f) OxS-score: control 2.2 ± 0.18, Tai Chi 0.88 ± 0.15, and walking 1.2 ± 0.12, *P* < 0.001.

In Figure 2, we show the particular markers of OxS adjusted to baseline conditions. Lipoperoxides (LPO) levels were lower in the subjects who practiced Tai Chi compared to the controls, with a borderline statistical significance (*P* = 0.08). Likewise, LPO levels were significantly higher in the walking group compared to the Tai Chi group (*P* < 0.05). Superoxide dismutase activity was significantly higher in the subjects who practiced Tai Chi compared to the controls (*P* < 0.05), while GPx activity was higher in the walking group compared to the control group with a borderline statistical significance (*P* < 0.07). The OxS-score was significantly lower in the Tai Chi and walking groups in comparison with control group (*P* < 0.001), although the change was more evident in the Tai Chi group. 

## 4. Discussion

Physical exercise has been recognized as part of a healthy lifestyle since ancient times, and the influence of physical exercise on the biology of aging is complex. It has also been demonstrated that the practice of moderate intensity physical exercise has an impact on various aspects of aging, including the notable development of an antioxidant response achieved by an adaptive process through mechanisms such as hormesis, in which continuous exposure to low quantities of stressors activates a controlled and beneficial response [8, 20–22].

Walking and Tai Chi are two moderate physical exercise modalities that are widely recommended for the aged. Walking has been suggested as a popular exercise that is accessible, can be performed freely, and can be incorporated into the activities of daily life and be continued into old age [23, 24]. Both the American College of Sports Medicine and the American Heart Association have indicated walking as an adequate alternative for older people because it is a safe activity that does not require special equipment and whose speed can be adjusted as a function of the individual physical condition in order to gradually achieve the required conditioning [9].

Numerous systematic reviews have demonstrated that walking programs have a positive influence on various health indicators including blood pressure, HDL cholesterol, proportion of body fat, aerobic capacity, mental health, and bone density [25–27]. 

On the other hand, Tai Chi is a traditional Chinese form of exercise, based on modifications of different martial arts. Tai Chi is classified as a moderate physical exercise, as its intensity does not exceed 55% of maximum oxygen consumption and 60% of maximum individual maximum heart rate. Recently, this exercise has increased in popularity because of evidence of the beneficial aspects of this discipline on the various aspects of health. The health benefits are particularly notable in older adults, and its practice does not pose risks, as it is a series of gentle and continuous motions that require control of position, deep breathing, and coordination of movements [10, 28].

The results of the present study, particularly the examination of routine biochemical parameters, indicate that Tai Chi has a positive effect on lipid metabolism. We observed a significant reduction in total cholesterol and in the LDL cholesterol fraction compared with the controls. These results are in accordance with earlier reports that have noted a reduction in total cholesterol, the LDL fraction, and an increase in HDL in various populations after the practice of Tai Chi [29, 30]. 

The physiological mechanisms that lead to these effects have not been entirely explained; however, the most accepted model suggests that it may be a consequence of the increase in metabolism produced by a chronic increase in muscular activity, as the movements, despite being gentle and low impact, require the holding of particular positions, which strengthen the muscle. In addition, these movements are combined with the demand for the expenditure of energy and the degradation of substrates produced by respiration [29–31]. 

Also, an effect synergy between the process of physical adaptation and the reduction of the dominance of the sympathetic nervous system given the increase in the vagus nerve response has been proposed as physiological mechanisms. The stimulus of the vagus nerve response is considered to be a result of the physiological and psychological relaxation associated with the practice of Tai Chi [28–30]. 

In the walking group, we did not observe any significant change in the routine biochemical parameters, which could be due to the length of the intervention. Some studies have noted beneficial effects on blood pressure and blood lipids following a longer intervention period [23, 31].

When considering the OxS markers, we observed a significant increase in SOD activity in the Tai Chi group in comparison with the control group, as well as a increase in the GPx activity in the walking group. These results are consistent with results reported by other studies, in which an increase in the antioxidant defense system after the regular performance of moderate physical exercise is often noted [8, 20, 32].

We observed an increase in GPx activity in the walking group versus the control group. Again, these results are consistent with what has been previously reported in other studies. The increase in GPx activity represents a modification that has been noted to be a component of the response of the glutathione system, which is indispensable in maintaining the redox state of the cell [33–35]. In this case, the redox state was altered by a substantial increase in reactive species (evident in this group through the increase of LPO) as a result of the increase in basic aerobic physical exercise such as walking, but it was not observed in the other study group, in which the exercise was aerobic, which could be part of a global response induced by the moderate exercise performed [36].

In the Tai Chi group, SOD activity increased significantly, which may be attributed to the stimulus involved in the constant performance of the exercise. A similar result has been previously reported after 12 months of the Tai Chi practice in adult subjects. Obtaining this response after only six months in our study may be due to the fact that Tai Chi was performed at least four times a week, while in the other studies, it was practiced less frequently [14].

It is important to note that OxS is a dynamic process in which observed modifications should be considered in the context of the entire response [4]. Accordingly, we examined the OxS index in the study subjects and found that value was significantly higher in controls versus the two intervention groups. These results suggest that moderate physical exercise *per se* has an antioxidant effect. Nevertheless, this effect is significantly enhanced when Tai Chi is practiced. The measured LPO levels in the Tai Chi group were significantly lower, and GAP levels were higher, which indicates an increase in the effectiveness of the antioxidant system to control the oxidative challenge represented by Tai Chi. Ultimately, this effectiveness was reflected in the index value of OxS, which was lower in this group.

It has been suggested that the mechanism that explains these findings may relate to an adaptive process influenced by the change in the body's redox balance in favor of more alkaline conditions in the cell. The reactive species generated during physical exercise act as the signal that is necessary for the activation of the MAPK proteins (p38 and ERK1/ERK2), which in turn activate the transcription factor sensitive to the redox state, NF-*κβ*, via activation of the kinase that phosphorylates the inhibitor of this factor (I*κβ*). Once freed of its inhibitor, NF-*κβ* migrates toward the nucleus where it can promote the synthesis of various antioxidant enzymes such as MnSOD, iNOS, and glutamylcysteine synthetase (GCS) [34]. These enzymes possess binding sites for this factor in the promoter region of their respective genes. An increase in promoter binding is globally manifested as an increase in the antioxidant response consequent to the moderate oxidative stimulus caused by the intensity of physical exercise [34, 37, 38]. 

The positive effects of Tai Chi may be due to a combination of various mechanisms, including the signaling mentioned previously. In addition to being a moderate form of exercise, it provides a relaxing psychological effect, similar to that reported for transcendental meditation. The practice of deep meditation (which is an essential characteristic of Tai Chi) is associated with lower levels of LPO, which has been explained as an effect of lower activity in the sympathetic nervous system. It has been demonstrated that the contrary effect, psychosocial stress, is accompanied by an increase in catecholamines and prostaglandins, which have been associated with an increase in LPO. Another hypothesis suggests that profound meditation increases levels of the hormone dehydroepiandrosterone, a marker for aging that has been linked with an increase in the activity of antioxidant enzymes. These possibilities, independent of but along with the mechanisms induced by the physical activity directly, allow us to suggest that the practice of Tai Chi promotes an antioxidant response [39–42]. 

## 5. Conclusions

The findings of our study suggest that the practice of Tai Chi generates a more intense antioxidant effect than walking, which could be linked with delaying the process of aging. However, these results need to be corroborated by long-term cohort studies. 

## Figures and Tables

**Table 1:** Age and blood pressure and body mass index by group.

	Control *n* = 23	Tai Chi *n* = 31	Walking *n* = 43
Age (years)	66.4 ± 4	66.7 ± 3.8	66.8 ± 3.9
SBP (mm/Hg)			
Baseline	121 ± 23	117 ± 12	127 ± 15
Postintervention	124 ± 10	111 ± 13*	123 ± 13
DBP (mm/Hg)			
Baseline	77 ± 12	74 ± 8	76 ± 9
Postintervention	75 ± 5	74 ± 10	75 ± 8
BMI (kg/m^2^)			
Baseline	27 ± 3	28 ± 5	28 ± 5
Postintervention	27 ± 4	27 ± 5	27 ± 6

SBP: systolic blood pressure; DBP: diastolic blood pressure; BMI: body mass index. ANOVA test, one time, *P = 0.001.

**Table 2:** Biochemical parameters of the study population by group.

	Control *n* = 23	Tai Chi *n* = 31	Walking *n* = 43
Glucose (mg/dL)			
Baseline	119 ± 50	107 ± 20	112 ± 21
Postintervention	104 ± 29	92 ± 12	90 ± 19
Cholesterol (mg/dL)			
Baseline	207 ± 44	201 ± 48	220 ± 44
Postintervention	219 ± 48	180 ± 41*	218 ± 40
Triglycerides (mg/dL)			
Baseline	144 ± 60	170 ± 115	171 ± 72
Postintervention	146 ± 51	155 ± 93	168 ± 63
HDL (mg/dL)			
Baseline	55 ± 19	50 ± 12	49 ± 8
Postintervention	53 ± 11	49 ± 12	50 ± 11
LDL (mg/dL)			
Baseline	122 ± 38	116 ± 45	137 ± 40
Postintervention	137 ± 43	99 ± 37**	135 ± 65

ANOVA test, one time. *P < 0.05, **P < 0.001 HDL: High-Density Lipoproteins, LDL: Low-Density Lipoproteins.

**Table 3:** Oxidative stress markers baseline and postintervention by group.

	Control *n* = 23	Tai Chi *n* = 31	Walking *n* = 43
Lipoperoxides (*µ*mol/L)			
Baseline	0.280 ± 0.07	0.287 ± 0.01	0.244 ± 0.01
Postintervention	0.297 ± 0.10	0.257 ± 0.09*	0.330 ± 0.01**
SOD (U/mL)			
Baseline	171 ± 15	169 ± 8	176 ± 7
Postintervention	171 ± 8	178 ± 9*	177 ± 9
GPx (U/L)			
Baseline	7381 ± 2694	6225 ± 2869	7756 ± 2697
Postintervention	8154 ± 4208	8410 ± 4084	11600 ± 6779**
TAS (mmol/L)			
Baseline	1.07 ± 0.23	0.82 ± 0.28	0.95 ± 0.3
Postintervention	1.03 ± 0.19	1.04 ± 0.16	1.01 ± 0.13
SOD/GPx			
Baseline	25 ± 12	34 ± 20	26 ± 13
Postintervention	37 ± 28	37 ± 27	16 ± 15
OxS-score			
Baseline	1.1 ± 0.1	2.6 ± 1.1	1.8 ± 1.4
Postintervention	2.0 ± 0.9	0.9 ± 0.8**	1.1 ± 0.8**

ANOVA test, one time. *P < 0.05, **P < 0.001, SOD: superoxide dismutase, GPx: glutathione peroxidase, TAS: Total antioxidant status, GAP: Antioxidant Gap, OxS-score: Oxidative Stress score.
